# Immune microenvironment changes induced by neoadjuvant chemotherapy in triple-negative breast cancers: the MIMOSA-1 study

**DOI:** 10.1186/s13058-021-01437-4

**Published:** 2021-05-26

**Authors:** Victor Sarradin, Amélie Lusque, Thomas Filleron, Florence Dalenc, Camille Franchet

**Affiliations:** 1 Department of Medical Oncology, Institut Claudius Regaud, Institut Universitaire du Cancer de Toulouse, IUCT-Oncopole, 1 avenue Irène Joliot-Curie, 31059 Toulouse Cedex 9, France; 2 Department of Biostatistics, Institut Claudius Regaud, Institut Universitaire du Cancer de Toulouse, IUCT-Oncopole, Toulouse, France; 3 Department of Pathology, Institut Claudius Regaud, Institut Universitaire du Cancer de Toulouse, IUCT-Oncopole, Toulouse, France

**Keywords:** Triple-negative breast cancer, Neoadjuvant chemotherapy, Immune microenvironment, Tumor-infiltrating lymphocytes, TILs, PD-L1, TIM-3, LAG-3, Immune checkpoint

## Abstract

**Background:**

The immune microenvironment (IME) of triple-negative breast cancers (TNBCs) and its modulation by neoadjuvant chemotherapy (NACT) remain to be fully characterized. Our current study aims to evaluate NACT-induced IME changes and assess the prognostic value of specific immune biomarkers.

**Methods:**

Tumor-infiltrating lymphocytes (TILs) were identified from hematoxylin-eosin-stained sections of paired pre- and post-NACT tumor samples from a TNBC cohort (*n* = 66) and expression of PD-L1, TIM-3, and LAG-3 evaluated by immunohistochemistry.

**Results:**

Overall TIL counts and PD-L1 expression did not differ pre- and post-NACT, but there was a response-specific statistically significant difference. TIL counts decreased in 65.5% of patients who achieved a pathological complete response (pCR) and increased in 56.8% of no-pCR patients (*p* = 0.0092). PD-L1 expression was significantly more frequently lost after NACT in pCR than in no-pCR patients (41.4% vs 16.2%, *p* = 0.0020). TIM-3 positivity (≥ 1%) was significantly more frequent after NACT (*p* < 0.0001) with increases in expression levels occurring more frequently in no-pCR than in pCR patients (51.4% vs 31%). LAG-3 expression significantly decreased after NACT, but there was no difference between response groups. Before NACT, a high TIL count (> 10%) was significantly associated with better overall survival (OS), *p* = 0.0112. After NACT, PD-L1 positivity and strong TIM-3 positivity (≥ 5%) were both associated with significantly worse OS (*p* = 0.0055 and *p* = 0.0274, respectively). Patients positive for both PD-L1 and TIM-3 had the worst prognosis (*p* = 0.0020), even when only considering patients who failed to achieve a pCR, *p* = 0.0479.

**Conclusions:**

NACT induces significant IME changes in TNBCs. PD-L1 and TIM-3 expression post-NACT may yield important prognostic information for TNBC patients.

**Supplementary Information:**

The online version contains supplementary material available at 10.1186/s13058-021-01437-4.

## Background

Neoadjuvant chemotherapy (NACT) is increasingly used to treat early-stage triple-negative breast cancers (TNBCs), and NACT response, in particular, the extent of residual disease post-NACT, yields important prognostic information [1, 2]. NACT treatment responses differentiate two groups of patients: those who achieve a pathologic complete response (the pCR group) and have a good prognosis and those with residual invasive disease (the no-pCR group) and a high risk of relapse. Patients in the no-pCR group are then either treated with adjuvant capecitabine [3] or considered for inclusion in a clinical trial.

Several TNBC studies have highlighted the association of tumor-infiltrating lymphocytes (TILs) with a good prognosis [4–13] and with a higher pCR rate after NACT [4–8, 13–18]. Although TIL counts are standardized [19, 20] and routinely assessed, they currently do not contribute to treatment strategy.

In addition to increased TIL counts, the expression of immune checkpoint (ICP) programmed death ligand 1 (PD-L1) appears to be associated with a higher pCR rate [14, 21–25] and better disease-free survival (DFS) [4]. Other novel immunological breast cancer (BrCa) targets, such as T-cell immunoglobulin and mucin domain-containing molecule 3 (TIM-3) [26, 27] and lymphocyte activation gene 3 (LAG-3) [28, 29], may yield additional prognostic information in TNBCs.

Although preclinical evidence supports that the anti-tumor activity of anthracyclines and alkylating agents may be partially mediated through the modulation of the anti-tumor immune response [30–32], only few studies have addressed this issue in TNBCs [33]. Identifying a potential impact of NACT on the TNBC immune microenvironment (IME) may help optimize the design of future clinical adjuvant trials for no-pCR patients and may lead to the development of novel biomarkers.

Our study’s primary objective was to evaluate IME changes induced by NACT in TNBCs. The prognostic value of immune biomarkers (TILs, PD-L1, TIM-3, and LAG-3) in terms of overall survival (OS) and their association with pathological response were also evaluated as part of the secondary objectives.

## Methods

### Population

We retrospectively identified 212 TNBCs out of a total of 666 early BrCa patients treated with NACT between June 2012 and October 2018 in our Comprehensive Cancer Center. TNBC was defined as estrogen and progesterone receptor expression < 10% and negative HER2 status. Only patients with available formalin-fixed paraffin-embedded (FFPE) samples from pre-NACT diagnostic biopsies as well as post-NACT surgical samples were included in the study (*n* = 66). Clinico-pathological, treatment, and follow-up data were extracted from patients’ medical records. The study was approved by the competent ethical committee. Written informed consent was obtained from patients.

### Pathology assessments

pCR was defined as the absence of residual invasive cancer cells both in the breast (ypT0 or ypTis) and the axilla (ypN0).

For patients who achieved pCR, we evaluated immune biomarkers in the tumor bed area. One representative paraffin block per case has been used for the study. This representative paraffin block was chosen after a comprehensive review of all slides of each case in relation to the macroscopic report. Lymphocytic infiltrates in non-carcinomatous lesions and normal breast structures were disregarded.

Stromal TIL scores were determined from hematoxylin-eosin-stained tissue sections and defined as the percentage of tumor stromal area comprising mononuclear inflammatory cells, according to the International Immuno-Oncology Biomarker Working Group guidelines for assessment before [19] and after NACT [20].

The following antibodies were used to characterize the immune infiltrate by immunochemistry on whole tissue section: PD-L1 clone SP142 (Ventana Medical Systems), TIM-3 clone AF2365 (R&D Systems), and LAG-3 clone NBP1-85781 (Novus Biologicals) dilution 1:500 Dako Flex (Dako).

Immune cell PD-L1 SP142 staining was quantitated (IC score) using a 4-class grading system, according to the manufacturer’s recommendations. The proportion of tumor area occupied by PD-L1 expressing tumor-infiltrating immune cells was scored as follows: IC0 = < 1%; IC1 = 1–4%; IC2 = 5–9%; IC3 = ≥ 10%.

TIM-3-positive cells were quantified as the proportion of stained cells in stromal regions. LAG-3-positive cells were reported as the number of stained cells in 10 high-power fields (× 400 magnification) in the strongest staining areas. For these two biomarkers, only moderate to strong staining intensity was considered as positive. Both cytoplasmic/membranous and paranuclear dot-like staining in mononuclear immune cells were quantitated.

We applied the following thresholds and cutoffs to score the number of TILs (cutoff > 10% or > 30%) and PD-L1 (negative = IC0 versus positive = IC1, IC2, and IC3), LAG-3 (cutoff > 0 or ≥ 10), and TIM-3 (cutoff ≥1% or ≥5%) staining patterns. TIM-3 was only scored as positive if it was ≥ 1% and LAG-3 when it was > 0. The number of TILs, LAG-3, and TIM-3 were also considered as continuous variables in the statistical analysis.

We used a composite criterion to identify patients who were PD-L1 positive and strongly TIM-3 positive (≥ 5%). We refer to this population as PD-L1+/TIM-3+.

### Statistical analysis

Categorical variables are represented as frequencies and percentages. Quantitative variables are expressed as medians and ranges (min:max). Comparisons between groups were performed using the chi-square test or Fisher’s exact test for categorical variables and the Mann-Whitney test for quantitative variables. Comparisons between the level of immune markers before and after NACT were performed using the Wilcoxon signed-rank test for paired data for quantitative variables and the McNemar test for categorical variables. For each continuous immune marker, the change post-NACT was determined as the difference in expression level before and after NACT.

Overall survival (OS) was defined as the time from surgery to death from any cause or the last follow-up (censored data). Survival curves were estimated using the Kaplan-Meier method. Univariable analyses were performed using the log-rank test for categorical variables and the Cox proportional hazard model for quantitative variables.

All statistical tests were two-sided, and a *p* value < 0.05 was considered to be statistically significant. Statistical analyses were performed using the STATA software version 16 (StataCorp LLC, College Station, TX).

## Results

### Patient characteristics

Our study included 66 patients who all received sequential NACT treatments. None of our patients received adjuvant capecitabine. Twenty-nine patients (43.9%) achieved pCR (pCR group) and 37 did not (56.1%; no-pCR group). Patient characteristics and comparisons between pCR and no-pCR groups are shown in Supplementary Table 1.

### Immune microenvironment before neoadjuvant chemotherapy

Table 1 presents immune microenvironment marker expression levels before and after NACT. Before treatment, the median TIL count was 10% (range 1.0:95.0) and 10 patients (15.2%) had a TIL count of > 30%. PD-L1, TIM-3, and LAG-3 were positive in 35 (53.0%), 21 (31.8%), and 51 (77.3%) patients, respectively. Nine patients (13.6%) exhibited strong TIM-3 staining (≥ 5%). Seventeen patients (25.8%) exhibited strong LAG-3 staining (≥ 10 positive cells).

**Table 1:** Description of the IME characteristics before and after neoadjuvant chemotherapy in the entire population (comparison with paired-test)

		Before chemo (*n* = 66) N (%)	After chemo (*n* = 66) N (%)	
TILs (%)	Median	10.0	10.0	*p* = 0.5598
	(Range)	(1.0:95.0)	(1.0:70.0)	
TILs	0–10%	36 (54.5%)	35 (53.0%)	*p* = 0.8474
	> 10%	30 (45.5%)	31 (47.0%)	
TILs	0–30%	56 (84.8%)	58 (87.9%)	*p* = 0.5930
	> 30%	10 (15.2%)	8 (12.1%)	
PD-L1	Negative (IC0)	31 (47.0%)	38 (57.6%)	*p* = 0.1936
	Positive (IC1/2/3)	35 (53.0%)	28 (42.4%)	
TIM-3 (%)	Median	0.0	1.5	***p*** **< 0.0001**
	(Range)	(0.0:15.0)	(0.0:20.0)	
TIM-3	< 1%	45 (68.2%)	21 (31.8%)	***p*** **< 0.0001**
	≥1%	21 (31.8%)	45 (68.2%)	
TIM-3	0–4%	57 (86.4%)	44 (66.7%)	***p*** **= 0.0093**
	≥5%	9 (13.6%)	22 (33.3%)	
PD-L1 and TIM-3	Others	62 (93.9%)	54 (81.8%)	***p*** **= 0.0209**
	PD-L1+/TIM-3+	4 (6.1%)	12 (18.2%)	
LAG-3 (%)	Median	2.5	3.0	***p*** **= 0.0389**
	(Range)	(0.0:37.0)	(0.0:23.0)	
LAG-3	0	15 (22.7%)	11 (16.7%)	*p* = 0.3173
	> 0	51 (77.3%)	55 (83.3%)	
LAG-3	0–9	49 (74.2%)	58 (87.9%)	***p*** **= 0.0126**
	≥10	17 (25.8%)	8 (12.1%)	

LAG-3 was the only immune biomarker significantly associated with pCR. The pCR rate in LAG-3-positive and LAG-3-negative patients was 53% and 13.4%, respectively (*p* = 0.0066) (Suppl. Table 2).

### Immune changes induced by chemotherapy

**Fig. 1:**
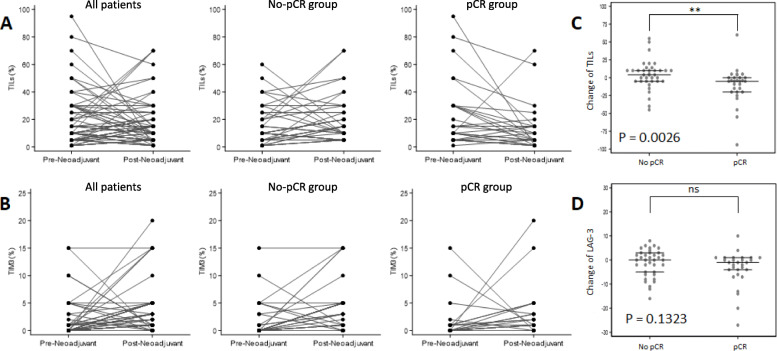
Changes of the tumor immune microenvironment induced by neoadjuvant chemotherapy. **a**, **b** Changes from biopsy (pre-neoadjuvant) to surgical tissue sample (post-neoadjuvant) for each patient, in all cases (left), in the no-pCR (middle), and in the pCR groups (right), for tumor-infiltrating lymphocytes (TILs) and TIM-3, respectively. **c**, **d** Comparison of changes from pre-neoadjuvant to post-neoadjuvant (as % change) between the no-pCR and the pCR groups, for TILs and LAG-3, respectively

Post-NACT, the median TIL count for the entire cohort remained unchanged at 10% (range 1:70) (Table 1). However, the change in TIL count differed significantly as a function of the NACT response (*p* = 0.0026), with a median change of − 5 (range of change − 94:60) in the pCR group and + 4 (range of change − 45:55) in the no-pCR group (Suppl. Table 3 and Fig. 1a–c). After NACT, there was a statistically significant difference between the number of TILs scored in the pCR and no-pCR groups (median (range) post-NACT: 5% (1:70) in the pCR group and 15% (5:70) in the no-pCR group, *p* = 0.0062). After NACT, 8 patients (12.1%) had > 30% TILs (6 in the no-pCR and 2 in the pCR group) (Suppl. Table 2).

There was no significant difference in PD-L1 expression pre- and post-NACT in the entire cohort, with 42.4% (*n* = 28) patients PD-L1 positive and 57.6% (*n* = 38) PD-L1 negative after NACT (*p* =0 .1936) (Table 1). This is in contrast with the large and significant difference detected between the pCR and no-pCR groups after chemotherapy, with 23 no-pCR patients (62.2%) and only 5 pCR patients (17.2%) PD-L1 positive after NACT (*p* = 0.0002) (Suppl. Table 2).

There was a significant increase in TIM-3 expression after chemotherapy: TIM-3 was negative in 21 (31.8%) and positive in 45 (68.2%) patients (*p* < 0.0001). After chemotherapy, 22 patients (33.3%) exhibited strong TIM-3 staining (≥ 5%) (*p* = 0.0093) (Table 1). This increase was observed in both groups but was significantly greater in the no-pCR patient group, with 45.9% of no-pCR patients staining strongly (≥ 5%) after NACT, compared to 17.2% of patients in the pCR group (*p* = 0.0141) (Suppl. Table 2 and Fig. 1b).

After NACT, 12 patients (32.4%) were PD-L1+/TIM-3+ in the no-pCR and none in the pCR group (*p* = 0.0007) (Suppl. Table 2).

LAG-3 expression significantly decreased after NACT (*p* = 0.0389) (Table 1), with a median change of − 0.5 (range of change − 27:10) (Suppl. Table 3), but this change was not significantly different in the pCR and no-pCR groups (*p* = 0.1323) (Fig. 1d).

**Fig. 2:**
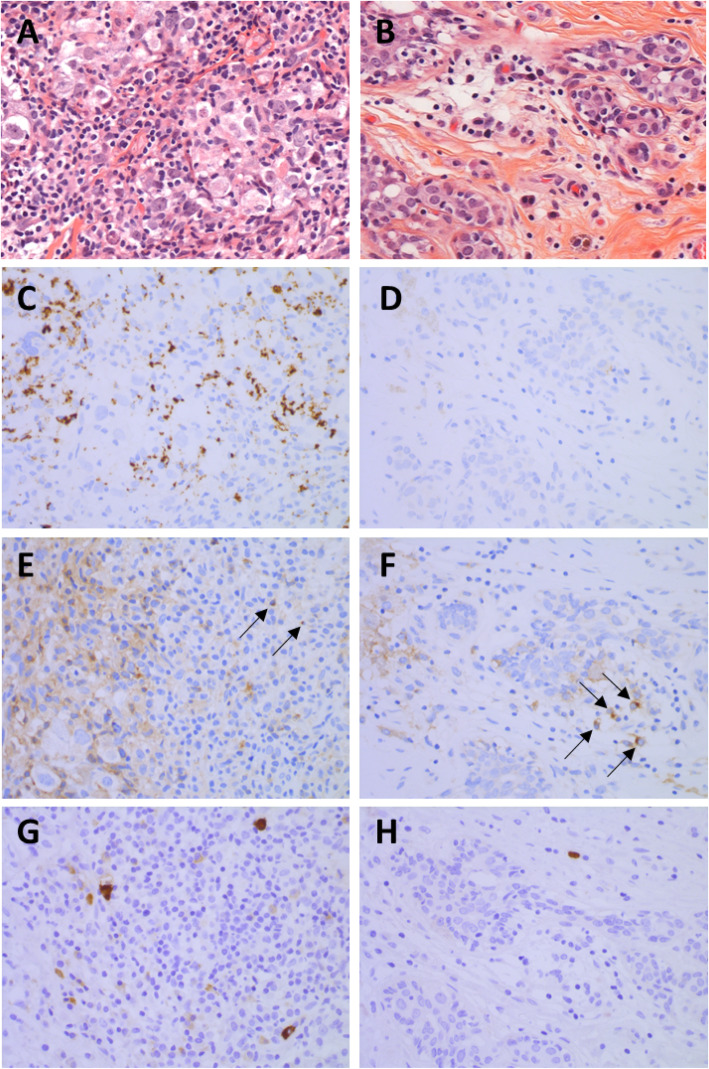
Representative images of changes of the immune microenvironment induced by neoadjuvant chemotherapy in one patient who achieved pCR and was still in remission 5 years after the surgery (H&E; PD-L1, TIM-3, and LAG-3 immunohistochemistry: × 400 magnification). To note that the different pictures presented here were not performed at the same site of the tissue section, and comparison between the localization of the different staining must not be carried out. **a** Baseline H&E of a significant TIL infiltrate (70%), indicative of a good prognosis and predictive of pCR. **b** Post-chemotherapy H&E staining showing a significant decrease in TILs but remaining high (15%). **c** Strong PD-L1 staining before treatment (IC2). **d** PD-L1 staining became negative after chemotherapy (IC0). **e** TIM-3 staining before treatment was negative (< 1%). **f** TIM-3 after chemotherapy became strongly positive (5%). **g** Strong LAG-3 staining before treatment (score = 26). **h** LAG-3 staining decreased after chemotherapy (score = 6)

Representative images of immunochemistry staining pattern changes are shown in Fig. 2.

### Association of immune biomarker expression and patient outcome

After a median follow-up of 35.4 months [95% CI 26.5–44.4], 13 patients died (19.7%).

**Fig. 3:**
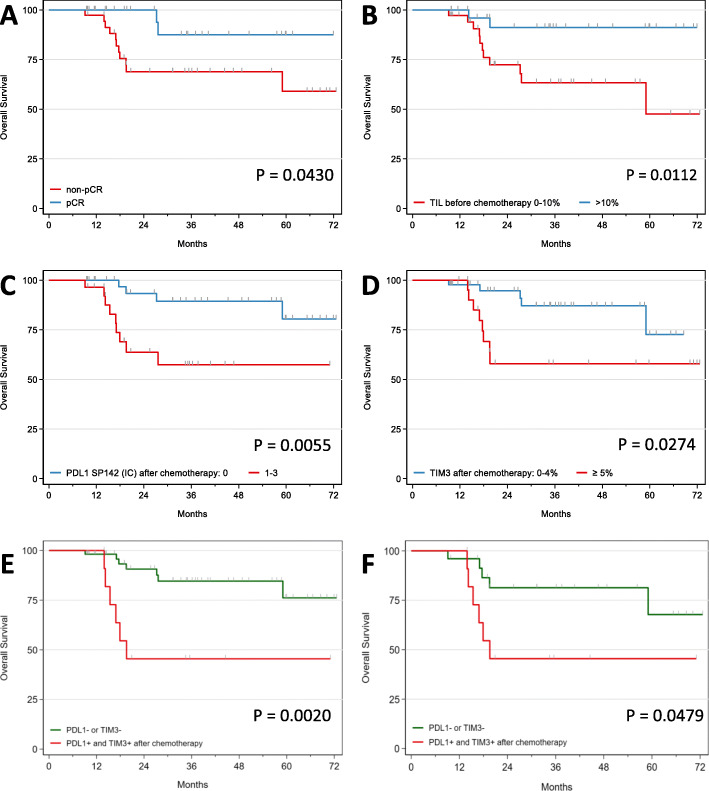
Kaplan-Meier curves of overall survival (OS) based on pathologic complete response (pCR) after neoadjuvant chemotherapy (NACT) (**a**), TIL counts before NACT (**b**), PD-L1 positivity after NACT (**c**), strong TIM-3 expression after NACT (**d**), PD-L1 positivity and strong TIM-3 expression after NACT (**e**), and PD-L1 positivity and strong TIM-3 expression after NACT in the no-pCR group (**f**)

Overall survival at 3 years (3yr-OS) was 76.3% [95% CI 61.8–85.9], with a significantly improved OS of patients in the pCR group compared to the no-pCR group (87.5% vs 68.9%, *p* = 0.0430) (Fig. 3a). Evidence of tumor cell vascular invasion in the surgical tissue samples was a strong indicator of poor prognosis (3yr-OS 33.3% vs 80.7%; *p* < 0.0001) (Suppl. Table 4).

#### Immune biomarkers before chemotherapy

The only significant prognostic factor associated with a good prognosis was a TIL count of > 10%, compared to patients with TIL counts ranging from 0 to 10% (3yr-OS 91.2% vs 63.4%, *p* = 0.0112) (Fig. 3b). For the 10 patients with > 30% TILs, the 3yr-OS was 100%. PD-L1 positivity tended to be associated with a better prognosis (3yr-OS 88.4% vs 63.8%) but did not reach statistical significance (*p* = 0.1111) (Suppl. Table 4). When evaluated as a continuous variable, TIL counts were associated with a better OS with a hazard ratio (HR) at 0.95 (0.89–1.00), but this was not statistically significant (*p* = 0.063). No association with OS was seen with TIM-3 (HR 1.00, *p* = 0.999) and LAG-3 (HR 0.98, *p* = 0.534), as continuous variables (Suppl. Table 5).

#### Immune biomarkers after chemotherapy

The association between an increased TIL count and a good prognosis was lost after chemotherapy.

PD-L1-positive staining and strong TIM-3 positivity were both significantly associated with poor prognoses (3yr-OS 57.4% vs 89.4%, *p* = 0.0055 and 58.0% vs 87.1%, *p* = 0.0274, respectively) (Fig. 3c, d). Patients with both PD-L1+/TIM-3+ tumors had significantly poorer prognoses (3yr-OS 45.5% vs 84.6%, *p* = 0.0020) (Fig. 3e).

When evaluated as a continuous variable, TIM-3 was associated with a poorer OS with a HR at 1.10 (1.00–1.22), which was close to be statistically significant (*p* = 0.051). No association with OS was seen with TIL counts (HR 0.98, *p* = 0.394) and LAG-3 (HR 1.00, *p* = 0.979), as continuous variables (Suppl. Table 5).

Interestingly, patients who were PD-L1 negative on biopsy but became positive after NACT had a worse prognosis (3yr-OS 19.9%, *n* = 11) (data not shown).

In the no-pCR group, the impact of positive PD-L1 and TIM-3 staining on OS was similar but no longer significant (3yr-OS 58.0% vs 84.6%, *p* = 0.1186 and 3yr-OS 50.0% vs 88.7%, *p* = 0.0702, respectively) (Table 2). However, dual PD-L1 and TIM-3 positivity identified a no-pCR subpopulation that correlated with poor prognoses. Indeed, PD-L1+/TIM-3+ no-pCR patients had significantly worse survival outcomes (3yr-OS 45.5% vs 81.3%, *p* = 0.0479) (Fig. 3f).

**Table 2:** Overall survival of the whole cohort at 3 years by surgical specimen (after chemotherapy) immune biomarker expression and overall survival for the subgroup of patients that failed to achieve pCR (no-pCR group)

	Evt/N	3yr-OS	95% CI	
All patients				
PD-L1				***p*** **= 0.0055**
Negative (IC0)	4/38	89.4%	70.6–96.5	
Positive (IC1/2/3)	9/28	57.4%	33.2–75.5	
TILs				*p* = 0.6312
0–10%	6/35	79.6%	57.0–91.2	
> 10%	7/31	72.5%	50.6–85.9	
TIM-3				*p* = 0.0840
< 1%	2/21	93.8%	63.2–99.1	
≥ 1%	11/45	67.0%	48.0–80.5	
TIM-3				***p*** **= 0.0274**
0–4%	5/44	87.1%	68.6–95.1	
≥ 5%	8/22	58.0%	33.1–76.4	
LAG-3				*p* = 0.7750
0	2/11	88.9%	43.3–98.4	
> 0	11/55	73.5%	56.9–84.5	
PD-L1 and TIM-3				***p*** **= 0.0020**
Others	7/54	84.6%	68.5–92.9	
PD-L1+/TIM-3+	6/12	45.5%	16.7–70.7	
No-pCR group				
PD-L1				*p* = 0.1186
Negative (IC0)	3/14	84.6%	51.2–95.9	
Positive (IC1/2/3)	8/23	58.0%	33.0–76.5	
TILs				*p* = 0.8008
0–10%	5/16	72.0%	41.1–88.6	
> 10%	6/21	65.7%	38.8–83.0	
TIM-3				*p* = 0.0702
0–4%	3/20	88.7%	61.4–97.1	
≥ 5%	8/17	50.0%	24.5–71.0	
PD-L1 and TIM-3				***p*** **= 0.0479**
Others	5/25	81.3%	57.3–92.6	
PD-L1+/TIM-3+	6/12	45.5%	16.7–70.7	

In the no-pCR group, immune biomarkers evaluated as continuous variables were not statistically associated with OS (Suppl. Table 5).

Patients in the no-pCR group who were PD-L1 negative on core biopsy but became positive after NACT also had poor prognoses (3yr-OS 29.2%, *n* = 8) (data not shown).

## Discussion

Our study not only examined changes in TIL counts and PD-L1 expression levels in 66 TNBC patients, before and after NACT, but is also, to the best of our knowledge, the first study to evaluate how changes in TIM-3 and LAG-3 expression levels correlate with pCR and OS.

Our study detected an overall cohort median TIL count of 10% at baseline, with 15.2% of TNBCs exhibiting greater than 30% TILs. These results are consistent with several reports in the literature [12–14], albeit that studies in the literature report higher median TIL counts [5, 7].

PD-L1 was expressed in 53% of cases, which is comparable to other reports which use the Ventana SP142 antibody [34, 35]. In our cohort, 32% and 77% of tumors expressed TIM-3 and LAG-3, respectively. Results obtained by Burugu et al. for TIM-3 (28% of TIM-3 positivity) are similar to ours [26]. However, the LAG-3 results reported by Burugu et al. and Bottai et al. (33% and 18%, respectively) differ from our results [28, 29]. This may be attributed to differences in the LAG-3 quantitation methodologies between studies. However, these differences may also reflect the very heterogeneous nature of the immune microenvironment in TNBCs which may support a rationale for immune checkpoint inhibitor and cytotoxic combination treatment to attempt to increase the pCR rate in this patient group [34–37].

TIL counts before NACT are both a strong prognostic factor for OS [4–13] and an established predictor of pCR [4–8, 13–18], which supports the notion that chemotherapy responses are at least partially immune mediated [30–33]. Our study did not detect any significant increases in baseline TIL counts of pCR patients, which may be attributed to a lack of power.

We report a trend of PD-L1 positivity before NACT correlating with an improved prognosis, but there was no association with pCR, perhaps due to our small sample size. This is in contrast to several other studies that report a significant association of positive PD-L1 staining with DFS [4] and pCR [14, 21–25].

Importantly, we show that positive baseline staining for LAG-3 but not for TIM-3 was associated with an increased probability of achieving pCR. We are, to the best of our knowledge, the first to report this finding. Larger studies will be required to confirm our observation.

Although several studies have previously reported changes in TIL counts before and after NACT and the prognostic significance of such changes [4, 5, 7, 12, 14], most of these studies did not specifically focus on TNBCs [12, 14]. The current literature therefore provides no definitive results regarding the prognostic value of TIL count changes in TNBCs. We report that a decrease in TIL count was significantly more frequently associated with pCR and conversely, that an increase in TIL count was more often associated with residual disease (Suppl. Table 3). We found that the number of TILs before (but not after) NACT was a significant prognostic marker of 3yr-OS, an association that persisted (close to significance) even when the pCR group was considered independently (data not shown). Our findings are consistent with observations reported by Castaneda et al. in 98 TNBC patients, of which 30% achieved pCR after NACT [7]. In a cohort of 72 TNBC patients, Dieci et al. observed an increase in TILs in residual disease after NACT which was associated with improved disease-free survival [4]. In a cohort of 104 TNBC patients, Lee et al. reported that compared to stable cases, a significant change, either a TIL count increase or decrease, between the pre-NACT biopsy and post-NACT residual tumor tissue sample, was associated with a better prognosis [5]. Despite the statistical significance of the association, this correlation may be attributed to the overrepresentation of cases with an increased post-NACT TIL count in the Lee et al. cohort. These data suggest that patients with increased TIL counts who do not achieve pCR after NACT may benefit from adjuvant ICP inhibitor therapy. Nonetheless, a better characterization of TIL levels is required to optimize therapeutic strategies in this patient group.

With regard to PD-L1 expression changes in TNBCs after NACT, Dieci et al. found a significant increase of PD-L1 expression in residual disease which was associated with an improved DFS of borderline statistical significance [4]. Pelekanou et al. detected decreased PD-L1 expression in a cohort encompassing all BrCa phenotypes and with no-pCR [12]. In another study, the same authors found that PD-L1 expression was not altered after NACT, but observed a 15% mean decrease in TILs [14]. We found no significant change in PD-L1 expression after NACT in our entire cohort, but an increase in PD-L1 expression in the no-pCR group. We also observed that PD-L1 positivity after NACT was significantly associated with poor OS in the entire cohort (*p* = 0.0055) and a trend of poor OS in the no-pCR group (*p* = 0.1186). The lack of standardized PD-L1 protocols makes it difficult to draw any conclusions from these studies and it is quite possible that the anti-PD-L1 VENTANA SP142 antibody we used identifies fewer PD-L1-positive patients [38].

Importantly, our study is the first to report changes in TIM-3 and LAG-3 expression levels before and after NACT in TNBCs and to determine their predictive and prognostic value. We found a significant increase in TIM-3 expression after NACT and strong TIM-3 expression post-NACT to be significant poor prognostic indicators in the entire population (*p* = 0.0274) and showed a similar trend in the no-pCR group (*p* = 0.0702). Interestingly, in the overall population as well as the no-pCR group, patients whose residual disease expressed both PD-L1 and TIM-3 had a worse prognosis (3yr-OS 45.5%).

These observations suggest that TNBCs remain immunogenic after NACT and may continue to be subject to antitumor immuno-surveillance, as evidenced by the increased infiltration of tumor lymphocytes in the no-pCR patient group. This hypothesis provides a rationale to explore reactivation of lymphocytes present in residual disease using adjuvant immunotherapy, such as in the current anti-PD-L1 antibody trial (NCT02954874). In light of strong TIM-3 expression being predictive of a good anti-PD-(L)1 immunotherapy response in a cohort of 30 head and neck cancers patients [39] and of anti-TIM-3 antibodies being evaluated alone or in combination with anti-PD-(L)1 therapy in several phase 1 trials [40], our own findings, should they be confirmed by larger multicentric studies, support the rational for testing immunotherapy adjuvant treatments specifically in patients that fail to achieve a pCR.

Our study is nevertheless limited by its small size and monocentric nature which reduces its statistical power and also limited more substantial subgroup and multivariate analyses. Moreover, relevant threshold for TIM-3 expression should be clarified in future studies.

## Conclusions

In conclusion, TNBCs have heterogeneous IMEs which are profoundly altered by NACT. After NACT, patients positive for both PD-L1 and TIM-3 had significantly worse prognoses and importantly this correlation persisted in the subgroup of patients that failed to achieve a pCR.

## Supplementary Information

**Additional file 1: Table S1.** Patients characteristics. Non-ductal histologic subtypes include one metaplastic chondroid (grade 2, no-pCR), one muco-epidermoid (grade 3, no-pCR) and one epidermoid metaplastic (grade 3, pCR). Abbreviations: D= docetaxel 100 mg/m^2^ (D1=D21), wP= weekly paclitaxel (80 mg/m^2^), (F)EC = 5-fluorouracil (500 mg/m^2^), epirubicin (100 mg/m^2^), cyclophosphamide (500 mg/m^2^). **Table S2.** Description of IME characteristics before and after chemotherapy, as a function of NACT response (pCR and no-pCR groups). Percentages for the pCR and no-pCR groups shown were calculated for each individual row. **Table S3.** Changes of IME characteristics induced by neoadjuvant chemotherapy, for the entire population, and as a function of the NACT response (pCR and no-pCR groups). **Table S4.** Overall survival at 3 years according to the initial clinical-pathological characteristics, as a function of core biopsy immune biomarkers (before chemotherapy) and vascular invasion evaluated on the surgical sample (after chemotherapy). **Table S5.** Association between overall survival (OS) and TILs, TIM-3 and LAG-3 evaluated as continuous variables.

## Data Availability

The datasets used and/or analyzed during the current study are available from the corresponding author on reasonable request.

## Abbreviations

IME: Immune microenvironment
TNBC: Triple-negative breast cancer
NACT: Neoadjuvant chemotherapy
TILs: Tumor-infiltrating lymphocytes
pCR: Pathological complete response
OS: Overall survival
ICP: Immune checkpoint
PD-L1: Programmed death ligand 1
DFS: Disease-free survival
BrCa: Breast cancer
TIM-3: T-cell immunoglobulin and mucin domain-containing molecule 3
LAG-3: Lymphocyte activation gene 3
