# Supplementary material for: Immune microenvironment changes induced by neoadjuvant chemotherapy in triple-negative breast cancers: the MIMOSA-1 study
Source: Breast Cancer Res. 2021 May 26;23:61. doi: 10.1186/s13058-021-01437-4 (PMC8157437; doi:10.1186/s13058-021-01437-4)
Supplement: Supplementary file 1 — Additional file 1: Table S1. Patients characteristics. Non-ductal histologic subtypes include one metaplastic chondroid (grade 2, no-pCR), one muco-epidermoid (grade 3, no-pCR) and one epidermoid metaplastic (grade 3, pCR). Abbreviations: D= docetaxel 100 mg/m2 (D1=D21), wP= weekly paclitaxel (80 mg/m2), (F)EC = 5-fluorouracil (500 mg/m2), epirubicin (100 mg/m2), cyclophosphamide (500 mg/m2). Table S2. Description of IME characteristics before and after chemotherapy, as a function of NACT response (pCR and no-pCR groups). Percentages for the pCR and no-pCR groups shown were calculated for each individual row. Table S3. Changes of IME characteristics induced by neoadjuvant chemotherapy, for the entire population, and as a function of the NACT response (pCR and no-pCR groups). Table S4. Overall survival at 3 years according to the initial clinical-pathological characteristics, as a function of core biopsy immune biomarkers (before chemotherapy) and vascular invasion evaluated on the surgical sample (after chemotherapy). Table S5. Association between overall survival (OS) and TILs, TIM-3 and LAG-3 evaluated as continuous variables. [file 13058_2021_1437_MOESM1_ESM.docx]

|  |  | Total  (n=66)  N(%) | No-pCR (n=37)  N(%) | pCR  (n=29)  N(%) |  |
| --- | --- | --- | --- | --- | --- |
| Age at biopsy | Median  (Range) | 52.0  (24.0:71.0) | 54.0  (32.0:71.0) | 48.0  (24.0:68.0) | p= 0.0757 |
| T | T1  T2  T3  T4 | 7 (10.6%)  29 (43.9%)  19 (28.8%)  11 (16.7%) | 2 (5.4%)  15 (40.5%)  11 (29.7%)  9 (24.3%) | 5 (17.2%)  14 (48.3%)  8 (27.6%)  2 (6.9%) | p= 0.1568 |
| N | N0  N1-N3 | 32 (48.5%)  34 (51.5%) | 15 (40.5%)  22 (59.5%) | 17 (58.6%)  12 (41.4%) | p= 0.1447 |
| Histology | Ductal  Other | 63 (95.5%)  3 (4.5%) | 35 (94.6%)  2 (5.4%) | 28 (96.6%)  1 (3.4%) | p= 1.0000 |
| Grade SBR | I or II  III  Missing | 12 (18.5%)  53 (81.5%)  1 | 10 (27.8%)  26 (72.2%)  1 | 2 (6.9%)  27 (93.1%)  0 | **p= 0.0310** |
| Chemotherapy | FEC  FEC or EC + wP  FEC +D  FEC or EC + D + wP | 1 (1.5%)  36 (54.5%)  26 (39.4%)  3 (4.5%) | 1 (2.7%)  18 (48.6%)  16 (43.2%)  2 (5.4%) | 0 (0.0%)  18 (62.1%)  10 (34.5%)  1 (3.4%) | p= 0.7214 |
| Surgery | Mastectomy  Conservative | 33 (50.0%)  33 (50.0%) | 22 (59.5%)  15 (40.5%) | 11 (37.9%)  18 (62.1%) | p= 0.0825 |
| Lymph nodes procedure | No  Dissection  Sentinel | 1 (1.5%)  48 (72.7%)  17 (25.8%) | 0 (0.0%)  29 (78.4%)  8 (21.6%) | 1 (3.4%)  19 (65.5%)  9 (31.0%) | p= 0.3193 |
| Radiotherapy | No  Yes | 3 (4.5%)  63 (95.5%) | 2 (5.4%)  35 (94.6%) | 1 (3.4%)  28 (96.6%) | p= 1.0000 |

**Supplementary table 1 -** Patients characteristics. Non-ductal histologic subtypes include one metaplastic chondroid (grade 2, no-pCR), one muco-epidermoid (grade 3, no-pCR) and one epidermoid metaplastic (grade 3, pCR).

Abbreviations: D= docetaxel 100 mg/m^2^ (D1=D21), wP= weekly paclitaxel (80 mg/m^2^), (F)EC = 5-fluorouracil (500 mg/m^2^), epirubicin (100 mg/m^2^), cyclophosphamide (500 mg/m^2^).

|  |  | Before chemotherapy | |  | After chemotherapy | |  |
| --- | --- | --- | --- | --- | --- | --- | --- |
|  |  | **No-pCR**  **(N=37)**  **N(%)** | **pCR**  **(N=29)**  **N(%)** |  | **No-pCR**  **(N=37)**  **N (%)** | **pCR**  **(N=29)**  **N (%)** |  |
| TILs (%) | Median  (Range) | 10.0  (1.0:60.0) | 10.0  (1.0:95.0) | p= 0.3523 | 15.0  (5.0:70.0) | 5.0  (1.0:70.0) | **p= 0.0062** |
| TILs | 0-10%  >10% | 21 (58.3%)  16 (53.3%) | 15 (41.7%)  14 (46.7%) | p= 0.6836 | 16 (43.2%)  21 (56.8%) | 19 (65.5%)  10 (34.5%) | p= 0.0719 |
| TILs | 0-30%  >30% | 32 (57.1%)  5 (50.0%) | 24 (42.9%)  5 (50.0%) | p= 0.7383 | 31 (83.8%)  6 (16.2%) | 27 (93.1%)  2 (6.9%) | p= 0.4496 |
| PD-L1 | Negative (IC0)  Positive (IC1/2/3) | 16 (51.6%)  21 (60.0%) | 15 (48.4%)  14 (40.0%) | p= 0.4932 | 14 (37.8%)  23 (62.2%) | 24 (82.8%)  5 (17.2%) | **p= 0.0002** |
| TIM-3 (%) | Median  (Range) | 0.0  (0.0:15.0) | 0.0  (0.0:15.0) | p= 0.9023 | 3.0  (0.0:15.0) | 1.0  (0.0:20.0) | **p= 0.0387** |
| TIM-3 | <1%  ≥1% | 26 (57.7%)  11 (52.3%) | 19 (42.3%)  10 (47.7%) | p= 0.6807 | 9 (24.3%)  28 (75.7%) | 12 (41.4%)  17 (58.6%) | p= 0.1398 |
| TIM-3 | 0-4%  ≥5% | 31 (54.3%)  6 (66.6%) | 26 (45.7%)  3 (33.4%) | p= 0.7203 | 20 (54.1%)  17 (45.9%) | 24 (82.8%)  5 (17.2%) | **p= 0.0141** |
| PD-L1 and TIM-3 | Others  PD-L1+/TIM-3+ | 34 (54.8%)  3 (75.0%) | 28 (45.2%)  1 (25.0%) | Not calculated | 25 (67.6%)  12 (32.4%) | 29 (100%)  0 (0.0%) | **p= 0.0007** |
| LAG-3 (%) | Median  (Range) | 2.0  (0.0:29.0) | 3.0  (0.0:37.0) | p= 0.1242 | 3.0  (0.0:23.0) | 3.0  (0.0:22.0) | p= 0.6189 |
| LAG-3 | 0  >0 | 13 (86.6%)  24 (47.0%) | 2 (13.4%)  27 (53.0%) | **p= 0.0066** | 6 (16.2%)  31 (83.8%) | 5 (17.2%)  24 (82.8%) | p= 1.0000 |
| LAG-3 | 0-9  ≥10 | 27 (55.1%)  10 (58.8%) | 22 (44.9%)  7 (41.2%) | p= 0.7899 | 32 (86.5%)  5 (13.5%) | 26 (89.7%)  3 (10.3%) | p= 1.0000 |

**Supplementary table 2 -** Description of IME characteristics before and after chemotherapy, as a function of NACT response (pCR and no-pCR groups). Percentages for the pCR and no-pCR groups shown were calculated for each individual row.

|  |  | Total  (n=66)  N (%) | No-pCR  (n=37)  N (%) | pCR  (n=29)  N (%) |  |
| --- | --- | --- | --- | --- | --- |
| TILs (%) | Median  (Range) | 0.0  (-94.0: 60.0) | 4.0  (-45.0: 55.0) | -5.0  (-94.0: 60.0) | **p= 0.0026** |
| TILs | Decrease  Stability  Increase | 31 (47.0%)  8 (12.1%)  27 (40.9%) | 12 (32.4%)  4 (10.8%)  21 (56.8%) | 19 (65.5%)  4 (13.8%)  6 (20.7%) | **p= 0.0092** |
| PD-L1 | Negative -> Negative  Positive -> Negative  Negative -> Positive  Positive -> Positive | 20 (30.3%)  18 (27.3%)  11 (16.7%)  17 (25.8%) | 8 (21.6%)  6 (16.2%)  8 (21.6%)  15 (40.5%) | 12 (41.4%)  12 (41.4%)  3 (10.3%)  2 (6.9%) | **p= 0.0020** |
| TIM-3 (%) | Median  (Range) | 1.0  (-15.0: 20.0) | 2.0  (-9.0: 15.0) | 0.0  (-15.0: 20.0) | **p= 0.0800** |
| TIM-3 ≥1% | Negative -> Negative  Positive -> Negative  Negative -> Positive  Positive -> Positive | 17 (25.8%)  4 (6.1%)  28 (42.4%)  17 (25.8%) | 7 (18.9%)  2 (5.4%)  19 (51.4%)  9 (24.3%) | 10 (34.5%)  2 (6.9%)  9 (31.0%)  8 (27.6%) | p= 0.3580 |
| LAG-3 | Median  (Range) | -0.5  (-27.0: 10.0) | 0.0  (-16.0: 8.0) | -1.0  (-27.0: 10.0) | p= 0.1323 |
| LAG-3>0 | Negative -> Negative  Positive -> Negative  Negative -> Positive  Positive -> Positive | 5 (7.6%)  6 (9.1%)  10 (15.2%)  45 (68.2%) | 3 (8.1%)  3 (8.1%)  10 (27.0%)  21 (56.8%) | 2 (6.9%)  3 (10.3%)  0 (0.0%)  24 (82.8%) | **p= 0.0090** |

**Supplementary table 3 -** Changes of IME characteristics induced by neoadjuvant chemotherapy, for the entire population, and as a function of the NACT response (pCR and no-pCR groups).

|  |  | Evt / N | 3yr-OS | 95% CI |  |
| --- | --- | --- | --- | --- | --- |
| T | T1/T2  T3/T4 | 6 /36  7 /30 | 78.5%  73.9% | 57.6-89.9  50.8-87.4 | p= 0.6735 |
| N | N0  N1-N3 | 4 /32  9 /34 | 82.4%  71.9% | 59.3-93.0  51.5-84.9 | p= 0.3466 |
| Grade SBR | I or II  III | 4 /12  9 /53 | 67.3%  77.6% | 27.7-88.5  61.0-87.8 | p= 0.2615 |
| Vascular invasion | No  Yes | 6 /47  5 /7 | 80.7%  33.3% | 61.6-90.9  4.6-67.6 | **p< 0.0001** |
| TILs | 0-10%  >10% | 11 /36  2 /30 | 63.4%  91.2% | 42.0-78.6  69.0-97.9 | **p= 0.0112** |
| TILs | 0-30%  >30% | 13 /56  0 /10 | 72.0%  100.0% | 55.6-83.2  NR | p= 0.0815 |
| PD-L1 | Negative (IC0)  Positive (IC1/2/3) | 9 /31  4 /35 | 63.8%  88.4% | 41.6-79.4  68.0-96.2 | p= 0.1111 |
| TIM-3 | <1%  ≥1% | 10 /45  3 /21 | 74.2%  81.4% | 55.9-85.8  52.6-93.6 | p= 0.4014 |
| TIM-3 | 0-4%  ≥5% | 11 /57  2 /9 | 76.8%  75.0% | 60.8-86.9  31.5-93.1 | Not calculated |
| LAG-3 | 0  >0 | 5 /15  8 /51 | 61.5%  81.4% | 30.8-81.8  64.5-90.8 | p= 0.1553 |
| LAG-3 | 0-9  >10 | 11 /49  2 /17 | 73.0%  85.1% | 55.1-84.6  52.3-96.1 | p= 0.3285 |

**Supplementary table 4 –** Overall survival at 3 years according to the initial clinical-pathological characteristics, as a function of core biopsy immune biomarkers (before chemotherapy) and vascular invasion evaluated on the surgical sample (after chemotherapy).

|  | Hazard Ratio (95% CI) | p-value |
| --- | --- | --- |
| Before chemotherapy (N= 66) | | |
| TILs | 0.95 [0.89; 1.00] | 0.063 |
| TIM-3 | 1.00 [0.85; 1.17] | 0.999 |
| LAG-3 | 0.98 [0.91; 1.05] | 0.534 |
| After chemotherapy, all patients (N=66) | | |
| TILs | 0.98 [0.94; 1.02] | 0.394 |
| TIM-3 | 1.10 [1.00; 1.22] | 0.051 |
| LAG-3 | 1.00 [0.90; 1.12] | 0.979 |
| After chemotherapy, No-pCR group (N= 37) | | |
| TILs | 0.97 [0.92; 1.03] | 0.319 |
| TIM-3 | 1.08 [0.97; 1.20] | 0.159 |
| LAG-3 | 1.02 [0.91; 1.15] | 0.729 |

**Supplementary table 5 –** Association between overall survival (OS) and TILs, TIM-3 and LAG-3 evaluated as continuous variables.
